# Combined effect of furanone fluconazole and amphotericin B against biofilms formed from Cryptococcus neoformans

**DOI:** 10.6026/97320630017536

**Published:** 2021-05-31

**Authors:** Kirishnamoorthy Meenakumari, Giridharan Bupesh

**Affiliations:** 1 Research & Development Wing, Sree Balaji Medical College and Hospital (SBMCH), Bharath University, BIHER, Chrompet, Chennai-600044, Tamil Nadu, India; 2 Department of Forest Science, Central University of Nagaland, Lumami, India

**Keywords:** Cryptococcus neoformans, fluconazole, amphotericin B, biofilm

## Abstract

It is of interest to document the combined effect of furanone fluconazole and amphotericin B against the biofilm formed by Cryptococcus neoformans. The MIC values of amphotericine B and Fluconazole were observed as 20µg/ml and 60µg/ml, respectively. The MIC for the Combination (Amphotericin B/ Fluconazole) was found to be at (15/20) µg/ml drug concentration. Thus, data shows the combined effect of furanone fluconazole and amphotericine B derivative against C. neoformans.

## Background:

Cryptococcus neoformans are the encapsulated fungus groups that cause meningoencephalitis in immuno-compromised individuals [1,3]. Cryptococcal spores and/or dried yeast cells are very small in size and can deposit deep in the respiratory tract following inhalation [2]. Amphotericin B is an antifungal drug given intravenously for serious fungal infections. Amphotericin B is effective with side effects to lungs and kidneys [4-10]. Polysaccharide capsule of C. neoformans enlarge the size of capsule during infection and the mechanism of growth was unknown [11- 13]. Therefore, it is of interest to document the combined effect of furanone fluconazole and amphotericin B against the biofilm formed by Cryptococcus neoformans.

## Materials and Method:

C. neoformans strain ATCC 14116 (isolated from pigeon dropping contaminated soil) was maintained in Potato Dextrose Agar (PDA) (Himedia-M096) slants and Potato Dextrose Broth (PDB) (Himedia- M403). This was inoculated at 37°C for 48 hours.

## Preparation of drug stock solutions:

20mg of amphotericin B and 20mg of fluconazole was weighted and dissolved in 1ml of Dimethyl sulfoxide (DMSO) and stored at 2°C in vials. Drug stock solution was diluted according to the culture and the DMSO concentration was equally maintained in all experiments (<1%) as shown in Table 1 (see PDF).

## Individual drug Treatment:

The MIC of antifungal drugs against C. neoformans biofilm is inoculated in a 96-well plate along with positive and negative control. This was incubated at 37°C for 7days. The 96 well plates are washed twice with Phosphate Buffer Saline (PBS) to remove free-living cells from the plate after the biofilm formation. Drugs were then added into the wells and incubated at 37°C for 48 hours. This was washed twice with PBS and the absorbance was measured using ELISA plate reader at 620nm.

## Combined drug treatment:

Two fold dilution method of the drug both above and below the MIC value were prepared. The individual MIC value of amphotericin B and Fluconazole is shown in Table 2 (see PDF). The antifungal drugs were serially diluted. Equal volume of inoculums was added using the Checkerboard method. The culture were added equally to the all wells and incubated at 37°C for 48 hours.

## Concentration of amphotericin B (µg/ml):

Amphotericin B was added in the concentration range of 0-5µg/ml. Fluconazole was added in the range of 0-25µg/ml. The plates are then incubated at 37°C for 48 hours. The incubation plates are washed twice with phosphate buffer saline (PBS) and the absorbance was measured using a ELISA plate reader at 620nm (Automatic ELISA Reader - Sunrise)

## Fluorescent Microscopy:

The cell were washed twice with PBS, the cells were stained with FITC and propidium Iodine that were prepared at the concentration of 30µg/ml respectively. The staining solutions were added and let to standing for 15min in dark. The staining solution was removed and washed with PBS. The samples were then analyzed using a Nikon Trinocular microscope (Nikon Eclipse Ni-U Japan) [14].

## Results and Discussion:

**Figure 1:**
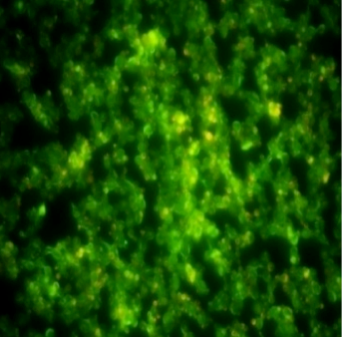
Fluorescent microscopy image of untreated melanized C. neoformans (ATCC strain) biofilm

**Figure 2:**
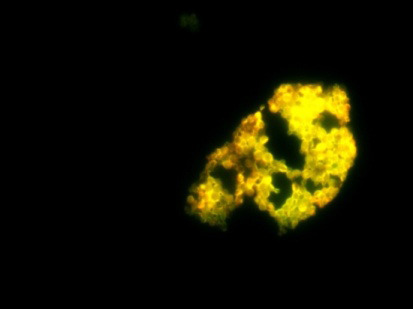
Fluorescent microscopy image of the combined treatment of melanized C. neoformans biofilm with the concentration of Amphotericin B and Fluconazole (15/20µg/ml)

**Figure 4:**
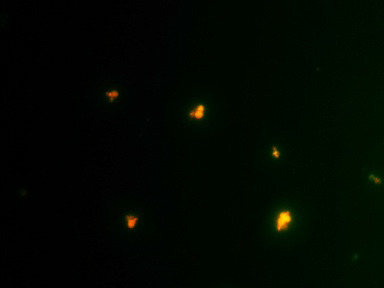
Fluorescent microscopy image of the combined treatment of melanized C.neoformans biofilm with the concentration of Amphotericin B and Fluconazole (20/20µg/ml)

The concentration range of Amphotericin B and Fluconazole used for ATCC strain was 16µg/ml to 240µg/ml and 60µg/ml to 100µg/ml. The MIC value was found to be 25/15 µg/ml (Amphotericin B/Fluconazole) (Figure 1). The MIC value in combination is slightly higher or equal to the individual drug MIC value. The concentration of Amphotericin B increased. This may be because of the resistance shown by the organism towards the single drug. MIC of amphotericin B and fluconazole were found to be 20µg/ml and 60µg/ml respectively. The MIC for the combination (amphotericin B/fluconazole) was found to be at (15/20) µg/ml drug concentration. The control of both the strains showing green fluorescent indicating live cells as shown in Figure 2-Figure 4. It was observed that the combination drug treatment is capable of disrupting the biofilm with killing the cells as well as disintegrate the biofilm. Biofilm formation has an important role for the C.neoformans to survive within the macrophages and to colonize in the central nervous system (CNS) [15]. Thus, disrupting the biofilm help to reducing the virulence nature to control the pathogen at the site of infection [16-17].

## Conclusion:

The data shows the combined effect of furanone fluconazole and amphotericine B derivative against C. neoformans by disrupting the formation of biofilms.

## Figures and Tables

**Figure 3:**
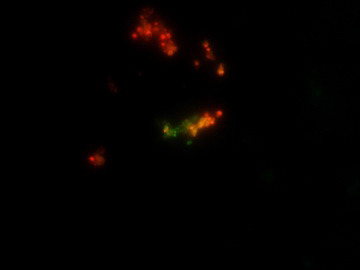
Fluorescent microscopy image of the combined treatment of melanized C. neoformans biofilm with the concentration of Amphotericin B and Fluconazole ((17.5/20µg/ml)
